# Pasteurized *Akkermansia muciniphila* improves irritable bowel syndrome-like symptoms and related behavioral disorders in mice

**DOI:** 10.1080/19490976.2023.2298026

**Published:** 2024-01-03

**Authors:** Maëva Meynier, Valentine Daugey, Geoffroy Mallaret, Sandie Gervason, Mathieu Meleine, Julie Barbier, Youssef Aissouni, Stéphane Lolignier, Mathilde Bonnet, Denis Ardid, Willem M. De Vos, Matthias Van Hul, Peter Suenaert, Amandine Brochot, Patrice D. Cani, Frédéric A. Carvalho

**Affiliations:** a NeuroDol, UMR 1107 INSERM, University of Clermont Auvergne, Clermont-Ferrand, France; b M2iSH, UMR 1071 INSERM, UMR1382 INRAé, University of Clermont Auvergne, Clermont-Ferrand, France; c Laboratory of Microbiology, Wageningen University, Wageningen, The Netherlands; d Human Microbiome Research Program, Faculty of Medicine, University of Helsinki, Helsinki, Finland; e The Akkermansia Company™, Mont-Saint-Guibert, Belgium; f Metabolism and Nutrition Research group, Louvain Drug Research Institute (LDRI), UCLouvain, Université Catholique de Louvain, Brussels, Belgium; g WELBIO-Walloon Excellence in Life Sciences and Biotechnology, WELBIO department, WEL Research Institute, Wavre, Belgium; h Institute of Experimental and Clinical Research (IREC), UCLouvain, Université catholique de Louvain, Brussels, Belgium

**Keywords:** Pasteurized *Akkermansia muciniphila*, IBS, colonic hypersensitivity, anxiety-like disorders, memory impairment, neuroinhibition

## Abstract

Gut – brain communications disorders in irritable bowel syndrome (IBS) are associated with intestinal microbiota composition, increased gut permeability, and psychosocial disturbances. Symptoms of IBS are difficult to medicate, and hence much research is being made into alternative approaches. This study assesses the potential of a treatment with pasteurized *Akkermansia muciniphila* for alleviating IBS-like symptoms in two mouse models of IBS with different etiologies. Two clinically relevant animal models were used to mimic IBS-like symptoms in C57BL6/J mice: the neonatal maternal separation (NMS) paradigm and the Citrobacter rodentium infection model. In both models, gut permeability, colonic sensitivity, fecal microbiota composition and colonic IL-22 expression were evaluated. The cognitive performance and emotional state of the animals were also assessed by several tests in the C. rodentium infection model. The neuromodulation ability of pasteurized A. muciniphila was assessed on primary neuronal cells from mice dorsal root ganglia using a ratiometric calcium imaging approach. The administration of pasteurized A. muciniphila significantly reduced colonic hypersensitivity in both IBS mouse models, accompanied by a reinforcement of the intestinal barrier function. Beneficial effects of pasteurized A. muciniphila treatment have also been observed on anxiety-like behavior and memory defects in the C. rodentium infection model. Finally, a neuroinhibitory effect exerted by pasteurized A. muciniphila was observed on neuronal cells stimulated with two algogenic substances such as capsaicin and inflammatory soup. Our findings demonstrate novel anti-hyperalgesic and neuroinhibitory properties of pasteurized A. muciniphila, which therefore may have beneficial effects in relieving pain and anxiety in subjects with IBS.

## Introduction

Disorders of gut – brain interaction (DGBIs) are a group of diseases characterized by chronic or recurrent gastrointestinal symptoms in the absence of underlying organic abnormalities.^1^ The best known DGBI is irritable bowel syndrome (IBS), characterized by chronic abdominal pain associated with altered bowel habits.^1^ Recent progress in better defining the underlying mechanisms in the pathogenesis of IBS could shift emphasis away from a symptom-based treatment paradigm to a more personalized therapeutic approach. Recent evidence has emerged of the importance of the role of the gut microbiota and its interactions with the gut-brain axis in the pathophysiology of IBS.^2^ Genetic background and mechanisms involving the local gastrointestinal tract, for example, in prior infectious enteritis, or an increase in gut permeability and psychosocial factors such as anxiety, depression and impaired cognition, are involved in its multifactorial pathogenesis.^3,4^ Pain is a key symptom of IBS and thus has a substantial impact on subject’s daily quality of life and, consequently, a significant economic impact on society. Finally, current treatments available to subjects with IBS are based on the reduction of symptoms. However, it has recently been proposed to individualize these treatments focusing on IBS pathophysiology and clinically identified biomarkers and not only symptoms.^5^

Various preclinical IBS models, mainly in rodents, have been developed to better understand the pathophysiology of IBS symptoms, mainly focusing on the colonic hypersensitivity. The stress induced by the neonatal maternal separation (NMS) paradigm is known to be an early traumatic experience that has long-term consequences on gastrointestinal functions.^6^ Specifically, the NMS paradigm has been shown to induce colonic hypersensitivity development associated with gut microbiota deviations in a subgroup of mice, making it a relevant non-inflammatory IBS model.^7^ Another pathophysiologic subtype of IBS is post-infectious IBS (PI-IBS) after a bacterial, protozoan, helminth, or viral gastrointestinal infection. Likewise, diarrhea-predominant IBS (IBS-D), PI-IBS is often characterized by an impaired intestinal or colonic permeability.^8^ Many of the gastrointestinal pathogens involved in PI-IBS are *Enterobacteriaceae*, such as *Salmonella spp*, *Shigella spp*, enterohemorrhagic *Escherichia coli* (EHEC), and enteropathogenic *E. coli* (EPEC). The murine model of *Citrobacter rodentium* infection is used to understand the pathophysiology associated with bacterial infections by EHEC and EPEC.^9,10^ Recently, this mouse model of *C. rodentium* infection was described as a relevant and predictive model to characterize the underlying mechanisms of PI-IBS.^9,11,12^ In recent years, the communication between the gut microbiota and the brain has generated considerable research activity which shows that further understanding of the disturbances of this microbiota-gut-brain axis is essential for the development of therapeutic strategies based on improving microbiota functions in the management of IBS.^13^

*Akkermansia muciniphila* is isolated as a mucus-degrading anaerobe^14^ and has been characterized as an abundant human colonic symbiont that colonizes the gut mucosa where it reinforces barrier function.^15,16^ The presence of this bacterium inversely correlates with body weight in rodents and humans.^15,17,18^ *A. muciniphila* plays a crucial role in the mutualism between the gut microbiota and host. Acting through different mechanisms, it is involved in the control of gut barrier function and other homeostatic and physiological functions.^16^ The mechanisms include the action of a heat-stable protein, called Amuc_1100, that signals the TLR2 receptor and increases barrier function by upregulating tight junction proteins, rationalizing why administration of pasteurized *A. muciniphila* has the same effects as live cells in improving barrier function.^19^ Subsequent studies confirmed and extended the beneficial effects of the oral administration of pasteurized *A. muciniphila* in several animal models. Daily administration of pasteurized *A. muciniphila* was found to counteract the development of obesity and related metabolic disorders in diet-induced obese mice.^19,20^ Additionally, in a proof-of-concept trial in obese subjects, it was found that pasteurized *A. muciniphila* compared to live cells induced a similar or in some cases greater improvement in clinical symptoms, such as glucose homeostasis, and lowering of serum levels of cholesterol, triglycerides, and LPS, the last of which reflected improved barrier function in the treated humans.^21^

Data from clinical studies with fecal microbiota transplantation^22^ or with probiotics^23^ in subjects with IBS have not yielded unequivocal results. Pasteurized *A. muciniphila* has demonstrated beneficial health effects and *A. muciniphila* was associated with alleviation of symptoms in subjects with IBS^24^, and so it was hypothesized that pasteurized *A. muciniphila* could have the potential to impact the gut-brain axis and alleviate symptoms in different IBS models. Hence, we decided to evaluate the cause–effect relationship of daily administration of pasteurized *A. muciniphila* in two preclinical IBS models with different etiologies: (1) the NMS paradigm mouse model, as a predictive model of non-inflammatory IBS, and (2) the *C. rodentium* infection mouse model, as a predictive PI-IBS model. The underlying mechanism of colonic hypersensitivity for abdominal pain relates to neuronal activation by either an agonist of the transient receptor potential vanilloid 1 (TRPV1) or G-protein-coupled receptors (GPCR), and therefore pasteurized *A. muciniphila* was tested in *in vitro* experiments with primary neuron cell lines. In this study, we show that pasteurized *A. muciniphila* improves IBS-like symptoms, related anxiety, and impaired cognition *in vivo* and hint to its underlying neuro-inhibitory potential *in vitro*.

## Results

### *Pasteurized* A. muciniphila *reduces colonic hypersensitivity induced in a non-inflammatory IBS mouse model*

**Figure 1.**
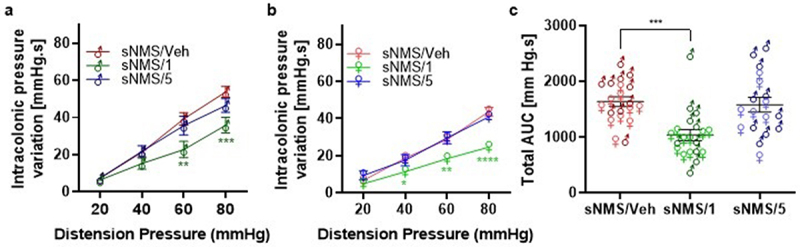
Effects of pasteurized *A. muciniphila* on colonic hypersensitivity induced in a non-inflammatory IBS mouse model. The sNMS mice that underwent a neonatal maternal separation paradigm and developed a colonic hypersensitivity, were treated for 10 days by gavage with a vehicle (sNms/veh) or with two different doses (3×10^9^ TFU for the sNMS/1 group or 6 × 10^8^ TFU for the sNMS/5 group) of pasteurized *A. muciniphila* (pAkk) (*n* = 10 males and *n* = 10 females per group). (a) Intracolonic pressure variation in response to a colorectal distension in male sNMS mice. (b) Intracolonic pressure variation in response to a colorectal distension in female sNMS mice. (c) Total area under the curve (AUC) for both male and female sNMS mice. Data are from two independent experiments. *p < 0,05; **p < 0.01; ***p < 0.001 sNms/veh vs. sNMS/1.

After applying the NMS paradigm during childhood, a first colonic distention (CRD) was performed on 8–10-week-old males and 10–12-week-old females in order to select mice with colonic hypersensitivity. From our two NMS experiments, we got 55,9% (62/111) and 56,3% (63/112) NMS mice with CHS that we called sensitized NMS (sNMS) mice (**Figure S1**). Thus, three sNMS mouse groups with similar colonic hypersensitivity were orally treated daily with two different doses of pasteurized *A. muciniphila*, 3 × 10^9^ (sNMS/1) or 6 × 10^8^ (sNMS/5) or with the vehicle (sNMS/Veh) for 10 successive days. One day after the last gavage, only mice treated with the highest dose of pasteurized *A. muciniphila* (sNMS/1) exhibited a significant decrease in colonic sensitivity assessed by CRD in comparison to the sNMS/Veh group. This effect is observed in male mice for the 60 and 80 mmHg distension pressure (Figures 1a and S2A and D), and in female mice for 40, 60, and 80 mmHg (Figures 1 and S2B and E). In addition, the distension volumes corresponding to each distension pressure were not different between groups, suggesting that colonic compliances are similar in our NMS mouse model. The total Area Under the Curve (AUC) over the entire distension ramp (from 20 to 80 mmHg) was calculated for each individual male and female mouse. This confirmed the significant reduction of the colonic hypersensitivity induced by the NMS paradigm only in sNMS mice treated with the highest dose of pasteurized *A. muciniphila* (sNMS/1) regardless of the sex of the animal (Figures 1c and S2C and F).

**Figure 2.**
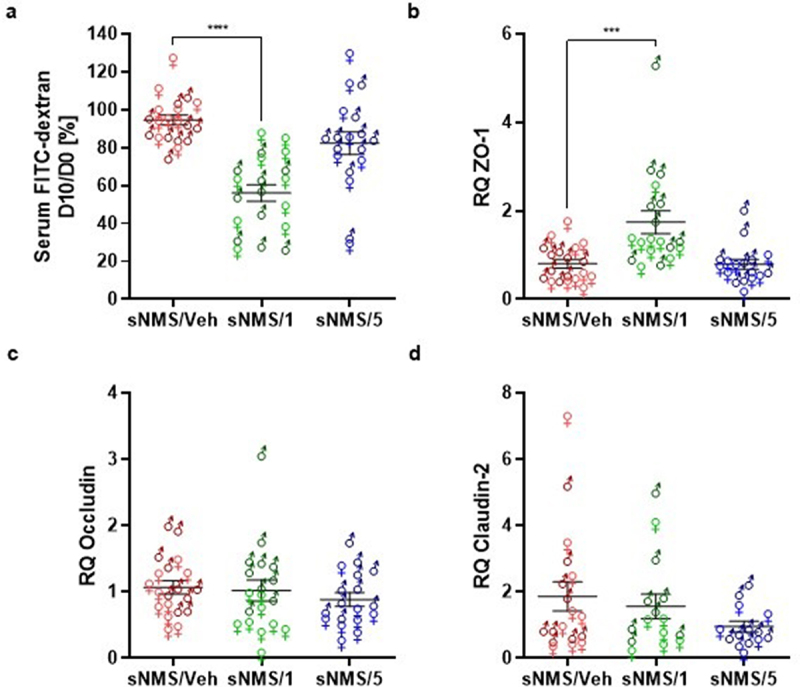
Effects of pasteurized *A. muciniphila* on intestinal permeability in a non-inflammatory IBS mouse model. The sNMS mice that underwent a neonatal maternal separation paradigm and have developed a colonic hypersensitivity, were treated for 10 days by gavage with a vehicle (sNms/veh) or with two different doses (3×10^9^ TFU for the sNMS/1 group or 6 × 10^8^ TFU for the sNMS/5 group) of pasteurized *A. muciniphila* (pAkk). (a) Intestinal permeability assessed by measuring 4 kDa FITC-Dextran concentration in serum, 3.5 hours after its administration by gavage. The percentage of intestinal permeability was calculated by doing the ratio between plasma FITC levels after treatment and before vehicle or pasteurized *A. muciniphila* treatment for each mouse (*n* = 10 males and *n* = 10 females per group). (b-d) colonic expression of (b) *ZO-1*, (c) *Occludin* and (d) *claudin-2* mRNA in each mouse group was quantified by RT-qPCR. Data are from two independent experiments. ***p < 0.001; ****p < 0.0001.

Despite the fact that the NMS paradigm is described as a non-inflammatory IBS model, there is a slight increase in intestinal permeability.^25^ Thus, measurement of the intestinal paracellular permeability has been performed using FITC-dextran gavage before and after the different treatments (vehicles or two different doses of pasteurized *A. muciniphila* including males and females). Only the highest dose of pasteurized *A. muciniphila* (sNMS/1 group) significantly reduced intestinal paracellular permeability by 44,0%, in comparison to the vehicle (sNMS/Veh group) (Figure 2a). At the end of the experiment, after 10 days of treatment and right after performing the CRD, we measured the colonic mRNA levels of the tight junction proteins ZO-1, Occludin, and Claudin-2. The expression of ZO-1 was significantly increased in the colon of mice treated with the highest dose of pasteurized *A. muciniphila* (Figure 2b), whereas the Occludin and Claudin-2 mRNA expressions were not affected independently of the dose of pasteurized *A. muciniphila* treatment (Figures 2c,d).

### *Pasteurized* A. muciniphila *reduces colonic hypersensitivity induced in a PI-IBS mouse model*

**Figure 3.**
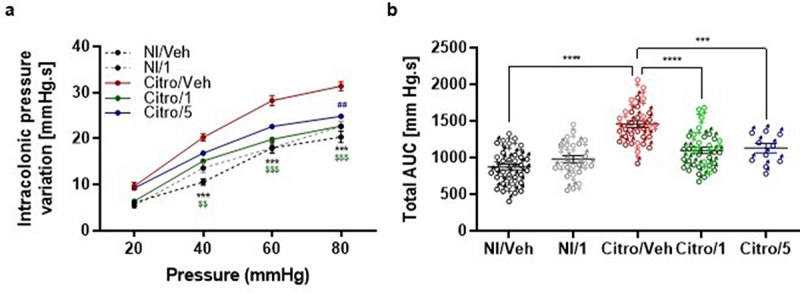
Effects of pasteurized *A. muciniphila* on colonic hypersensitivity induced in a post-infectious IBS mouse model. Mice were infected with *citrobacter rodentium* to induce a PI-IBS mouse model. The non-infected mice were inoculated with 200 µL of sterile PBS. During the post-infectious phase, mice were treated by gavage for 8 days from 16 days post infection (DPI) to 23 DPI. Non-infected (Ni/veh) and infected (Citro/Veh) mice were forced fed with the vehicle. Non-infected mice from the NI/1 group were forced-fed with 3 × 10^9^ TFU of pasteurized *A. muciniphila* (pAkk). Mice from the Citro/1 and Citro/5 group were forced-fed with 3 × 10^9^ TFU or 6 × 10^8^ TFU of pasteurized *A. muciniphila*. (a) Post-infectious colonic sensitivity assessed by measuring intracolonic pressure variations in response to a colorectal distension and (b) corresponding total area under the curve (AUC) (*n* = 10–34 per group). Data are from three independent experiments. * : Citro/Veh vs. NI/Veh. $ : Citro/Veh vs. Citro/1. # : Citro/Veh vs. Citro/5.

In the *C. rodentium* infection model of C57BL6/J mice, *C. rodentium* is completely cleared up from 16 days post-infection (DPI), thus marking the beginning of the post-infectious phase.^11^ Male mice were then treated every day by gavage with two different doses of pasteurized *A. muciniphila* for eight successive days. A significant colonic hypersensitivity was observed in the Citro/Veh mouse group, in comparison to the mice non-infected by *C. rodentium* and treated only with the vehicle (NI/Veh), for the 40, 60, and 80 mm Hg distension pressures (Figures 3a and S3A, C, and E). In addition, the distension volumes corresponding to each distension pressure were not different between groups, suggesting that colonic compliances are similar in our *C. rodentium* infection mouse model. The calculation of the total AUC over the entire distension ramp (from 20 to 80 mmHg) for each mouse confirmed the colonic hypersensitivity in the Citro/Veh group in comparison to the NI/Veh group (Figures 3b and S3B, D, and F). The treatment with the lowest dose of pasteurized *A. muciniphila* (Citro/5) significantly reduced the colonic sensitivity only for the 80 mm Hg pressure distension. In addition, the treatment with the highest dose of pasteurized *A. muciniphila* (Citro/1) significantly reduced the colonic sensitivity for the 40, 60, and 80 mm Hg pressure distension (Figures 3a and S3A, C, and E). In addition, treatments with both the lowest and the highest dose of pasteurized *A. muciniphila* significantly reduced the total AUC of the CRD responses in comparison to the Citro/Veh group (Figures 3b and S3B, D, and F). To ensure that the treatment with the highest dose of pasteurized *A. muciniphila* had no effect on animals with a normal level of colonic sensitivity, we performed similar experiments on a non-infected mouse group treated with the highest dose of pasteurized *A. muciniphila* (NI/1). Results confirmed the beneficial effect of pasteurized *A. muciniphila* on colonic sensitivity observed in infected mice. In contrast, pasteurized *A. muciniphila* treatment did not affect colonic sensitivity in non-infected control mice (Figures 3a,b and S3C-F).

**Figure 4.**
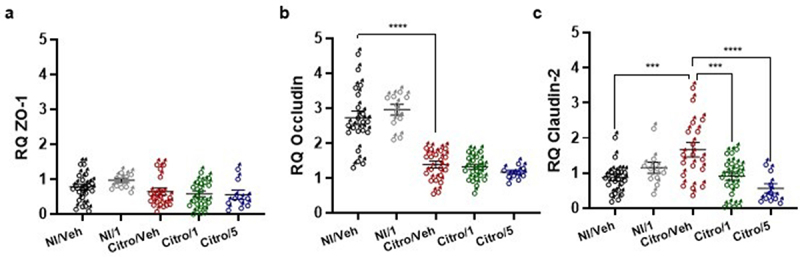
Effects of pasteurized *A. muciniphila* on intestinal tight junction protein expression in a post-infectious IBS mouse model. Mice were infected with *citrobacter rodentium* to induce a PI-IBS mouse model. The non-infected mice were inoculated with 200 µL of sterile PBS. During the post-infectious phase, mice were treated by gavage for 8 days from 16 days post infection (DPI) to 23 DPI. Non-infected (Ni/veh) and infected (Citro/Veh) mice were forced-fed with the vehicle. Non-infected mice from the NI/1 group were forced-fed with the highest dose (3×10^9^ TFU) of pasteurized *A. muciniphila* (pAkk). Mice from the Citro/1 and Citro/5 group were forced-fed with the highest dose (3×10^9^ TFU) or the lowest dose (6×10^8^ TFU) of pasteurized *A. muciniphila*. Colonic expression of (a) *ZO-1*, (b) *Occludin* and (c) *claudin-2* mRNA in each mouse group (*n* = 9–20 per group) was quantified by RT-qPCR. Data are from two independent experiments. *p < 0.05; **p < 0.01; ****p < 0.0001.

The mRNA expression of different tight junction proteins such as Occludin, Claudin-2, and ZO-1 was also measured. The *C. rodentium* infection model is known to reduce Occludin expression and to increase Claudin-2 expression^11^. Similar results were observed in the Citro/Veh group, regarding Occludin mRNA expression (Figures 4b) and Claudin-2 mRNA expression (Figures 4c), in comparison to the NI/Veh group, thereby showing the validity of the model. Occludin mRNA expression following *C. rodentium* infection was not reversed by any dose of pasteurized *A. muciniphila* treatment (Figures 4b). Conversely, pasteurized *A. muciniphila* treatments restored Claudin-2 mRNA expression to a similar level as in the NI/Veh mouse group (Figures 4c). Finally, there was no significant difference in ZO-1 mRNA expression in either group (Figures 4a).

### *Beneficial effects of pasteurized* A. muciniphila *on extra-intestinal IBS comorbidities in a PI-IBS mouse model*

**Figure 5.**
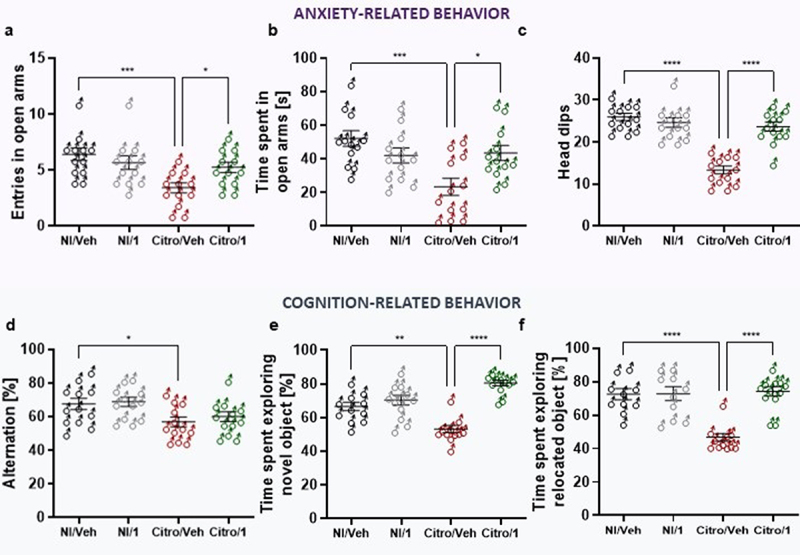
Pasteurized *A. muciniphila* treatment reverses post-infectious anxiety- and cognition-like behavior induced by *C. rodentium* infection. After a treatment with pasteurized *A. muciniphila* (pAkk), anxiety-related behavior induced by a *C. rodentium* post-infection were determined by EPM test at 21 days post-infection (DPI) and by the hole-board test at 22 DPI (*n* = 12 per group). (a) Number of entries and (b) time spent in open arms (monitoring for 5 minutes) during the EPM test. (c) Number of head dips made on the hole-board during 5 minutes were quantified (*n* = 10–12 per group). Cognition-related behaviors analyses were performed using (d) the Y-maze test in which spontaneous alternation were measured for 10 minutes and (e-f) time spent exploring the novel object and the relocated object during novel object and relocation tests, respectively. Data are from two independent experiments. *p < 0.05; **p < 0.01; ***p < 0.001; ****p < 0.0001.

At 21 DPI and after 5 days of treatment, the Elevated-Plus Maze (EPM) reference test was used to assess anxiety-like behavior in mice treated with the highest dose of pasteurized *A. muciniphila*. First, there was no difference in the number of entries in closed arms in either of the groups indicating there was no effect of the model nor of pasteurized *A. muciniphila* treatment on the locomotion capacity of the different mice. As previously described, the mice infected with *C. rodentium* (Citro/Veh group) exhibited anxiety-like behavior, as reflected by the significant decrease in the number of entries and time spent in the open arms compared to the NI/Veh mouse group (Figures 5a,b). In contrast, a significant increase in the time spent and the number of entries in the open arm were observed in the Citro/1 group compared to the Citro/Veh group (Figures 5a,b). To confirm that the daily treatment with the highest dose of pasteurized *A. muciniphila* reversed the anxiety-like behavior induced by the *C. rodentium* infection, the hole board test was used to make another anxiety-related behavior assessment. Mice from the Citro/1 group (infected mice treated with pasteurized *A. muciniphila*) made significantly more head dips than infected mice treated with the vehicle (Figure 5c). The results obtained with the two behavioral tests showed that pasteurized *A. muciniphila* is able to reverse anxiety-like behavior in the *C. rodentium* model of IBS after only 5 days of gavage.

Cognitive performance was then evaluated using the Y-maze test to assess working memory^26^ and the NOR/NLR behavioral test for learning and recognition memory.^27^ During the Y-maze assay, the percentage of alternation was significantly lower in the Citro/Veh mouse group than in the non-infected NI/Veh mouse group (Figure 5d). However, this parameter did not return to a normal spontaneous alternation in infected mice even after a treatment with pasteurized *A. muciniphila* (Figure 5d). The pattern of the cognitive impairment in the Citro/Veh mouse group was confirmed by NOR/NLR tests in which infected mice treated with vehicle alone spent less time exploring both novel object and relocated object than non-infected mice (Figures 5e,f). However, pasteurized *A. muciniphila* significantly improved time spent exploring both novel object and relocated object compared to non-infected mice (Figures 5e,f). To our knowledge, this is the first time that pasteurized *A. muciniphila* has been shown to have an effect on cognitive traits such as learning and recognition memory in the *C. rodentium* infection model of IBS.

### *Impact of pasteurized* A. muciniphila *on fecal microbiota composition and colonic IL-22 expression in IBS-like models*

**Figure 6.**
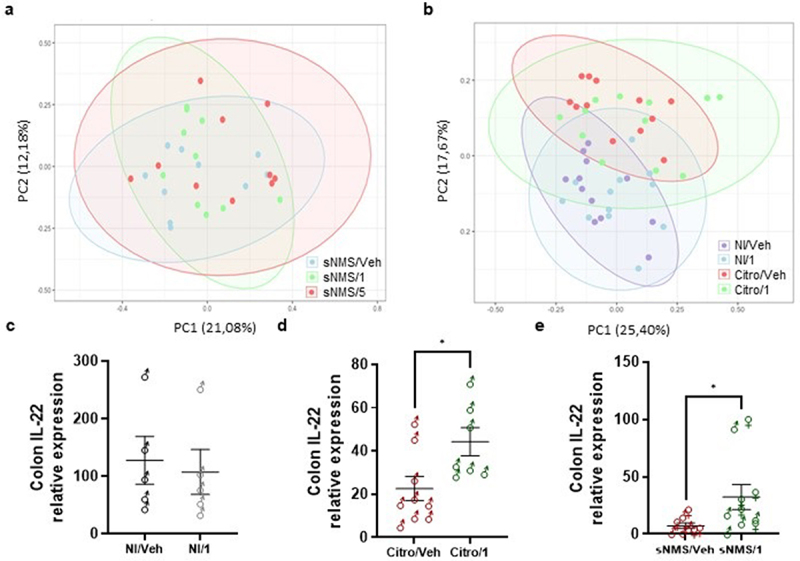
Impact of pasteurized *A. muciniphila* on fecal microbiota composition and colonic IL-22 expression in IBS-like models. (A and B) fecal microbiota composition was determined using 16S rRNA amplicon sequencing (V4 regions) (*n* = 10–12 per group), and principal coordinates analysis (PCoA) with a Bray-Curtis distance metric of the different mouse groups are shown for the NMS paradigm (a) and the *C. rodentium* infection (b) IBS-like mouse models. Ellipses represent the 95% confidence interval and the first and second components of the variance are shown in percentages. (e-e) colonic IL-22 expression (relative to 26S expression) in non-infected mice with or without 3 × 10^9^ TFU pasteurized A. muciniphila (pAkk) treatment (c), in C. rodentium infected mice with or without 3 × 10^9^ TFU pAkk treatment (d), and in sensitized NMS mice with or without 3 × 109TFU pAkk treatment (e). *p < 0,05; veh treatment vs. pAkk treatment.

Since gut microbiota is recognized as a key player in IBS physiopathology, we have performed a microbiota analysis in our two IBS-like mouse models (from the mice shown in the figures S2 A-C for the NMS model and in the figures S3 D-F for the *C. rodentium* infection model) to determine whether pasteurized *A. muciniphila* could modulate IBS-like symptoms though a microbiota modulation. Our results show that pasteurized *A. muciniphila* treatment has no significant impact on microbiota composition (Figures 6a,b). The taxonomic composition of the microbiota was also studied at the level of families. In the NMS experiment, no significant difference was found when comparing sNMS/Veh group with sNMS/1 group and sNMS/Veh group with sNMS/5 group after adjusting for multiple testing (False Discovery Rate [FDR] = 1%). In the *C. rodentium* infection model, several taxa were found to be differently abundant between non-infected mice (NI/Veh group) and mice infected with *C. rodentium* (Citro/Veh) as previously published^11^, but when comparing Citro/Veh and Citro/1, no taxa was found to be differently abundant when adjusting for FDR after multiple comparisons.

In a previous study, we showed that local IL-22 expression in the colon restored colonic sensitivity and normal animal behavior in the *C. rodentium* infection model of IBS.^11^ Thus, we investigated the impact of pasteurized *A. muciniphila* on colonic IL-22 expression using a very sensitive approach, the digital droplet PCR (ddPCR), to increase the detection sensitivity for colonic IL-22 expression. A slight increase in IL-22 colonic expression was observed after pasteurized *A. muciniphila* treatment only in both NMS and *C. rodentium* infection models, without reaching a level similar, however, to that observed in non-infected WT mice (Figures 6c–e).

### *Neuromodulatory potential of pasteurized* A. muciniphila *on nociceptors from dorsal root ganglia innervating the colon*

**Figure 7.**
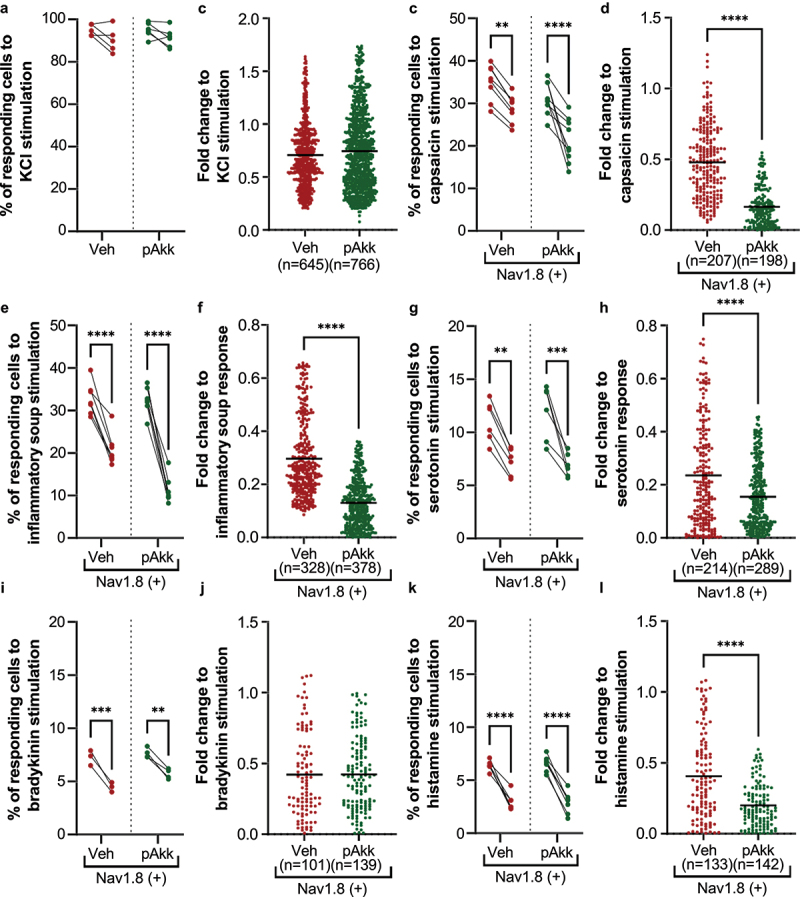
Neuromodulatory potential of pasteurized *A. muciniphila* on nociceptors from dorsal root ganglia innervating the colon. (a-b) neurons from all murine DRGs were activated with a pan-neuronal stimulation with KCl 30 mM. The percentage of neurons responding to the two KCl 30 mM simulations (a) and their activation level after the second KCl 30 mM stimulation (b) has been evaluated. (c-l) nociceptors (Nav1.8-cre-TdTomato positive neurons) from murine DRG innervating the colon were activated with several algogenic substances such as (c-d) capsaicin, (e-f) inflammatory soup, (G-H) serotonin 15 µM, (i-j) bradykinin 5 µM, or (k-l) histamine 15 µM. A ratiometric calcium imaging was used to visualize in real-time the neuronal activation level before and after incubation of pasteurized *A. muciniphila* (pAkk) or its vehicle. For the data analysis, the kinetic stimulation was studied for each viable neuronal cell. The percentage of neurons responding to each algogenic substances and the fold change in response intensity to the second stimulation (after vehicle or pasteurized *A. muciniphila* incubation) relative to the response intensity to the first stimulation of all viable neuronal cells belonging were calculated. Data are from two or three independent experiments. ****p < 0.0001.

Some *E. coli* strains, such as *E. coli* Nissle 1917, have analgesic effects by inhibiting the calcium flux induced by nociceptor activation in sensory neurons.^28,29^ A similar hypothesis was formulated and verified by performing similar experiments on murine DRG neurons activated by either an agonist of the calcium channel TRPV1 (capsaicin) or an inflammatory soup containing a mix of agonists (Serotonin, bradykinin, and histamine) for GPCRs involved in colonic hypersensitivity. Ratiometric calcium imaging was used to visualize neuronal activation levels in real-time before and after administration of pasteurized *A. muciniphila* or its vehicle incubation. Firstly, a pan-neuronal KCl (30 mM) stimulation was used to stimulate all neuronal cells from all mouse dorsal root ganglia (DRG). Results indicated that pasteurized *A. muciniphila* did not modify the percentage of neurons responding to KCl 30 mM stimulation (Figure 7a) or their activation level after the second KCl 30 mM stimulation (Figure 7b). Secondly, to test the effect of the pasteurized *A. muciniphila* on the activation of colonic nociceptors, neuronal primary cultures were performed using only mouse DRG innervating the colon from Nav1.8-Cre-TdTomato mice, so as to discriminate the nociceptors from non-nociceptive neurons. Thus, for the stimulation with algogenic substances, pasteurized *A. muciniphila* incubation significantly reduced by 2.90- and 2.28-fold the effect of the second stimulation by capsaicin (5 µM) or an inflammatory soup (IS), respectively, without affecting the percentage of responding neurons (Figures 7c–f). Finally, the different compounds of the IS were used individually to stimulate neuronal cells before and after pasteurized *A. muciniphila* incubation. The pasteurized *A. muciniphila* treatment significantly reduced by 1.52- and 2.03-fold the effect of the second stimulation by 15 µM of serotonin or 15 µM of histamine, respectively, but had no effect on the second stimulation by 5 µM bradykinin, without affecting the percentage of responding neurons (Figures 7g–l). Taken together, these results show that pasteurized *A. muciniphila* is capable of inhibiting calcium signaling in primary afferent DRG nociceptors, by directly or indirectly modulating the calcium channel TRPV1 or GPCRs, such as serotonin or histamine receptors.

## Discussion/Conclusion

Increased colonic sensitivity in IBS subjects is associated with the severity of gastrointestinal symptoms^30^, such as chronic abdominal pain, which is a key symptom in IBS subjects.^31^ Given the difficulty of controlling this symptom^32^, it is a major problem in human health that significantly impairs the quality of life of subjects suffering from IBS and also places an economic burden on health-care resources. Current treatments for chronic abdominal pain and associated anxio-depressive symptoms related to IBS have shown limited efficacy with often significant side effects.^33^ Disturbances in the microbiota-gut-brain communication axis in IBS, such as changes in the composition of the intestinal microbiota are well documented as are extra-intestinal factors like anxiety and depression symptoms.^34^ For example, a recent study demonstrated that the abundance of *A. muciniphila* is reduced in IBS and, in addition, could be responsible for abdominal pain relief after fecal microbiota transplantation in IBS subjects.^24^ Thus, we decided to evaluate the efficacy of the oral administration of *A. muciniphila* against IBS-like symptoms in two different mouse models. In the present study, we show that the administration of pasteurized *A. muciniphila* significantly reduced colonic hypersensitivity induced in both mouse models and was accompanied by a reinforcement of the intestinal barrier function. Beneficial effects of pasteurized *A. muciniphila* treatment were also evidenced in our experiments on anxiety-like behavior and memory defects induced in the PI-IBS model. These *in vivo* results combined with the *in vitro* neuromodulatory potential of pasteurized *A. muciniphila* characterized by calcium imaging would prompt the hypothesis that pasteurized *A. muciniphila* could be beneficial in alleviating the chronic abdominal pain as well as anxiety in subjects with IBS.

Equilibrium of the gut-brain axis depends primarily on intestinal barrier integrity. Adverse events such as early life traumatic experiences or episodes of gastroenteritis can weaken the intestinal barrier and lead to a defensive reaction by the host involving immune and inflammatory mediators, which in turn could be responsible for the triggering or development of a colonic hypersensitivity. One of the reference models for IBS associated with early life traumatic events is the NMS paradigm in rodents.^35^ Our NMS paradigm protocol did not induce colonic hypersensitivity in all adult mice unlike in other studies reported elsewhere.^7,36^ Thus, after the first CRD to identify mice with colonic hypersensitivity, only sensitized NMS (sNMS) mice treated with the highest dose of pasteurized *A. muciniphila* exhibited a significant decrease in the level of colonic hypersensitivity to a level comparable to that of non-handled mice (mice without NMS stress) or non-sensitized NMS mice, irrespective of sex. In our study, we also used another relevant preclinical model of PI-IBS based on an enterobacterial gastrointestinal infection with *C. rodentium*.^11^ As observed in our NMS-induced IBS model, pasteurized *A. muciniphila* treatment significantly affected colonic hypersensitivity by decreasing colonic sensitivity to a level comparable to that in non-infected mice, without modifying the basal colonic sensitivity in the non-infected mice. To date, a correlation link has been established between the presence of *A. muciniphila* in gut microbiota and colonic sensitivity only in a rat model^37^ or in clinical studies on IBS subjects.^24^ Thus, to our knowledge, our study is the first to describe the direct beneficial effect of pasteurized *A. muciniphila* on colonic sensitivity and to suggest that it could relieve chronic abdominal pain.

A defect in intestinal permeability is often associated with colonic hypersensitivity in preclinical models or with IBS symptoms in humans.^6,31^ Our results show a beneficial effect of the highest dose of pasteurized *A. muciniphila* on global *in vivo* intestinal permeability as detected by the FITC-dextran challenge assay. To further study intestinal permeability and to understand how pasteurized *A. muciniphila* treatment could reinforce the intestinal epithelial barrier, we measured in both IBS models the mRNA expression of three different tight junction proteins: Occludin, Claudin-2, and ZO-1. Despite the significantly lower overall intestinal permeability revealed by our FITC-dextran gavage assay, pasteurized *A. muciniphila* treatment differentially modulated the expression of these three tight junction proteins according to the used IBS model. The highest dose of pasteurized *A. muciniphila* increased the expression of ZO-1, which has been associated with a decrease in *in vitro* and *in vivo* permeability^11^ in the NMS-induced IBS model, whereas, in our *C. rodentium* PI-IBS model, a similar treatment restored the level of colonic expression of Claudin-2, but not Occludin.^11^ *A. muciniphila* has already been widely documented as being able to strengthen the intestinal barrier function in mice and humans by increasing the production of intestinal tight junctions, endocannabinoids, antimicrobial peptides, and endogenous enteric neuropeptides.^15,19,21^

There is growing recognition that bidirectional signaling between the gut and the brain contributes to extra-intestinal IBS comorbidities such as anxio-depressive symptoms and memory impairment.^38^ The gut-brain axis is a bidirectional communication axis involving different signaling metabolites (bile acids, short-chain fatty acids, cytokines, neurotransmitters) *via* the systemic and vagus nerve routes.^39^ Thus, the effect of pasteurized *A. muciniphila* was evaluated on extra-intestinal IBS comorbidities only in our *C. rodentium* PI-IBS model. Although many studies have shown behavior alterations in the NMS paradigm IBS-like mouse model, our NMS paradigm model never exhibited anxiety-like behavior after an EPM test or a hole board test. In addition, colonic hypersensitivity in our model was induced in only around 50% of mice, which we assume to be due to our NMS paradigm protocol being less severe and drastic than other published NMS protocols. There are wide variations in the implementation of these NMS protocols in the literature, including different separation times (3 h to 6 h), life periods of separation (from P1–16 to P1-P21), different methods of separation (all the litters together, litters individually separated, or in different rooms), and different growing environments.^40^ In our *C. rodentium* PI-IBS model, 5 days of treatment with pasteurized *A. muciniphila* corrected the anxiety-like behavior induced, as assessed by the EPM reference test, and confirmed by a second anxiety-like behavior test, the hole board test. Other cognitive-related behavioral tests were also performed and evidenced a beneficial effect of pasteurized *A. muciniphila* on episodic memory, as assessed by the novel object recognition (NOR) and the novel location recognition (NLR) tests, but treatment did not prevent spatial working memory loss, an index of short-term memory-loss, in *C. rodentium-*infected mice. Until recently, only improvements in anxiety-related behavior have been shown to coincide with increased relative abundance of *Akkermansia* in the gut microbiota composition.^41^ A very recent study by Sun *et al*. reported that *A. muciniphila* and its outer membrane protein Amuc_1100 can alleviate antibiotic-induced anxiety and depression by affecting the BDNF/TrkB signaling pathway.^42^ Another explanation for the effects of pasteurized *A. muciniphila* treatment on animal behavior, and also on colonic hypersensitivity, is its possible impact on IL-22 colonic expression. We have also shown that IL-22 colonic delivery alleviates IBS-like symptoms.^11^ In addition, it is already known that *A. muciniphila* interaction with the host involves TLR2-signaling pathways^19,43^ and modulation of IL-10 and IL-22 cytokines.^44,45^ Thus, a moderately induced colonic expression of IL-22 by pasteurized *A. muciniphila* would restore, at least in part, colonic sensitivity and normal animal behavior in our two IBS-like mouse models.

The development of a colonic hypersensitivity in animals or humans can involve the activation of pro-nociceptive neuronal receptors such as TRPV1^46,47^ or GPCRs, such as serotonin^48^, bradykinin,^49^ and histamine^50^ receptors. In addition, the activation of the TRPV1 channel can be potentiated by a large number of compounds including serotonin^51^, bradykinin,^52^ and histamine.^53^ We therefore hypothesized that pasteurized *A. muciniphila* treatment could directly modulate activation of the primary afferent fibers, alleviating colonic hypersensitivity in our *in vivo* IBS mouse models. Several electrophysiological recording approaches can be used to verify the contribution of peripheral pain mechanisms to the mechanism of action of pasteurized *A. muciniphila* treatment on colonic hypersensitivity-associated IBS symptoms, such as perforated patch clamp recordings from colonic DRG neurons or afferent nerve recordings from colonic afferents during CRD. In our study, we decided to use another approach based on neuron primary cultures from Nav1.8-Cre-TdTomato mouse DRG innervating the colon (from L6-S1 DRG) and to follow the activation of the neurons by calcium imaging, to investigate whether pasteurized *A. muciniphila* could directly act on the gut nervous system and in particular the colonic nociceptors involved in the modulation of pain perception or the transmission of the pain signal. Our results show that pasteurized *A. muciniphila* did not exert neural inhibition under pan-neuronal stimulation with KCl 30 mM, which suggests that a more specific mechanism could be involved in the interaction between pasteurized *A. muciniphila* and neuronal cells. However, a neuroinhibitory effect exerted by pasteurized *A. muciniphila* was observed on neuronal cells stimulated with two algogenic substances such as capsaicin and inflammatory soup solution, in addition to a tachyphylaxis effect. To our knowledge, no interaction between *Akkermansia* and the TRPV1 cation channel, which is activated by capsaicin, has been documented. However, capsaicin has been shown to reduce body weight mainly through activation of the TRPV1 channel and by promoting the abundance of gut bacteria, notably *A. muciniphila*. ^54–56^ Regarding the inflammatory soup challenge, the effect of pasteurized *A. muciniphila* on neuronal activation could be due to its well-known anti-inflammatory properties. Thus, neuron primary cultures from Nav1.8-Cre-TdTomato mouse DRG innervating the colon were separately challenged with serotonin, bradykinin, or histamine. A very strong effect was observed with serotonin and histamine stimulation, which opens up new fields of investigation into the interactions between pasteurized *A. muciniphila* and neuronal cells.

Chronic abdominal pain is an important problem in human health, which can lead to a significant impairment of well-being.^1^ Modifications at the level of the microbiota/gut/brain communication axis that trigger the appearance of anxiety-depressive symptoms have been described in chronic abdominal pain-associated pathologies such as IBS.^57^ Our study showed a significant effect of pasteurized *A. muciniphila* treatment on colonic hypersensitivity induced by two IBS mouse models. In addition, such beneficial effects are associated with significantly reduced intestinal permeability, and also to a significantly reduced anxiety-like behavior and memory impairment in the *C. rodentium* PI-IBS model. Finally, our study was based on the characterization of the neuromodulatory activity of pasteurized *A. muciniphila* bacteria in an attempt to find a new non-pharmacological approach to relieve chronic abdominal pain and anxio-depressive symptoms in IBS subjects.

## Materials and methods

### Animals and ethics statement

Male or female C57BL6/J mice (5–6 weeks old) were obtained from Janvier laboratories (Le Genest-Saint-Isle, France). They were housed in a temperature-controlled room (21 ± 1°C) under standard conditions, with access to water and food (from Safe, irradiated [>10 kiloGrays] food A04–10) *ad libitum*. The daylight cycle was from 6.00 am to 6.00 pm (on/off). NMS model mice were housed in a Specific-Pathogen-Free (SPF) animal facility at the University of Clermont Auvergne (Clermont-Ferrand, France). For the *C. rodentium*-infected mouse model, animals were housed in the Biosafety Level 2 (ABSL2) facility of the University of Clermont Auvergne (Clermont-Ferrand, France). All experiments were performed according to the ethical guidelines set out by the International Association for the Study of Pain^58^, complied with the European Union regulations, and were approved by ethics committees: the local committees C2EA–02 of Clermont-Ferrand (approvals NMS: protocol number CE110–12 and CE111–12; PI-IBS: protocol number EU0116–3460). The authors have read the ARRIVE guidelines, and the manuscript was prepared and revised according to the recommendations of the ARRIVE guidelines.

### IBS mouse models

#### NMS paradigm mouse model

One week after delivery of the mice, breeding cages were made to house one male and three females. The first litter of pups was not kept. The second litter was used to create separation groups as previously described.^25^ Briefly, pups were separated from their mother 3 h per day (from 9:00 a.m. to 12:00 p.m.) between day 2 and day 14 postnatal (P2 to P14). Pups were placed in a separate room from that in which they were bred. The mothers were not in the same room as the pups during the 3 h of separation. To avoid any problems arising from the temperature conditions, pups were placed on a warming blanket with cotton to keep them at 32°C and to prevent any bodily contact, they were separated into individual box (2 × 3 cm). After the last 3-h separation on day 14, pups were kept with their mother until weaning at day 21. In adulthood, a first colonic distension (CRD) was performed between W8–10 for males and W10–12 for females to identify the colonic hypersensitive mice, which were designated as sNMS mice. A NMS mouse is defined as sNMS when its AUC > mean + 2 × SD of NH mice AUC. The experimental protocol for the CRD is described below in colonic distension test.

#### PI-IBS mouse model

One week after acclimatization in our animal biosafety level A2 facility, mice were infected with *C. rodentium* to induce a PI-IBS mouse model as previously described.^11^ Briefly, *C. rodentium* strain (ATCC® 51459TM DBS100) was grown overnight at 37°C in Luria Broth (Dutscher, Issy-les-Moulineaux, France) without shaking. Mice were then orally infected with *C. rodentium* (1 × 10^9^ CFU/mice in 200 µL of PBS). Non-infected (NI) mice were inoculated with 200 µL of sterile PBS. At 16 DPI, fecal samples were plated on MacConkey agar (Dutscher, Issy-les-Moulineaux, France) to count the *C. rodentium* CFU to confirm its clearance.

### *Pasteurized* A. muciniphila *treatment*

Pasteurized *A. muciniphila* Muc^T^ (ATTC BAA-835) and placebo preparations were produced as described previously^21^ and administered by oral gavage to mice from the two IBS mouse models described above.

In the NMS paradigm mouse model, sNMS mice were housed together for at least 5 days before the beginning of the 10-day gavage treatment (at 4:00 p.m.). Mice on the NMS-vehicle group (NMS/Veh group) were force-fed with the vehicle (Sterile PBS containing 2.5% glycerol). Mice of the highest (NMS/1) and the lowest (NMS/5 group) dose were, respectively, forced-fed with 3 × 10^9^ Total Fluorescent Units (TFU) or 6 × 10^8^ TFU of pasteurized *A. muciniphila*. For each group, the volume administered was 250 µL in order to give all the animals the same volume during oral gavage.

In the PI-IBS mouse model, and after screening for resolution of *C. rodentium* infection (16 DPI), mice were treated by gavage for 8 days (at 4:00 p.m.) depending on their group. Non-infected (NI/Veh) and infected (Citro/Veh) mice were force-fed with the vehicle. Non-infected mice from the NI/1 group were force-fed with 3 × 10^9^ TFU of pasteurized *A. muciniphila*. Finally, mice from the Citro/1 and Citro/5 group were forced-fed with 3 × 10^9^ TFU or 6 × 10^8^ TFU of pasteurized *A. muciniphila*, respectively. For each group, the volume to be administered are 250 µL in order to give all the animals the same volume during oral gavage.

### Colorectal distension test

Colonic sensitivity was assessed by a noninvasive manometric method recently developed and validated in mice.^59^ This parameter was evaluated by quantifying intracolonic pressure variations in response to colorectal distension (CRD) with a miniaturized pressure transducer catheter (model 600; Millar Instruments, Houston, USA) equipped with a custom-made “balloon-pressure sensor” (1 cm wide × 2 cm long) prepared from a polyethylene plastic bag. On the day of the CRD experiment, mice were accustomed to the holding device for 1 h before the CRD. Then, mice were anesthetized with isoflurane (3% in O_2_). A polyethylene balloon with a connecting catheter was introduced into the rectum such that the distal end of the balloon was positioned at 1 cm from the anal margin. Subsequently, the animals were placed in homemade restriction cages, tape-maintained on the tail, and allowed to recover for 30 min before CRD to reduce motion artifacts caused by restraint stress. A polyethylene balloon coupled to a pressure sensor was connected to an electronic barostat (Distender Serie s II, G&J Electronics, Toronto, Canada) and a preamplifier (PCU-2000 Dual Channel Pressure Control Unit, Millar Instruments) connected to the PowerLab interface. The barostat was used to manage air infiltration in the distension balloon. It controlled balloon pressure during CRD experiment, and also it minimized any interference of colonic motor activity changes during balloon inflation. Intracolonic pressure variations in response to CRD were measured with the miniaturized pressure transducer catheter, whose signal was acquired and analyzed with LabChart 7 software (ADInstruments, Paris, France), to assess visceral pain-related responses. The “treated signal” trace of intracolonic pressure variations was extracted from the original “raw signal” recorded by the method previously described and was analyzed blindly.^60^

### In vivo *intestinal paracellular permeability*

*In vivo* intestinal paracellular permeability was assessed using fluorescein dextran (FITC-dextran 3000–5000 Da) (TdB Consultancy AB, Uppsala, Sweden) as previously described.^25^ Briefly, before and after the treatment period, mice were orally gavaged with 0.6 mg/g body weight of FITC-dextran (around 200 µL from a stock solution of 60 mg/mL of sterile PBS). Blood samples were then obtained from the retro-orbital venous plexus 3.5 h after this administration. Control mice (not treated with pasteurized *A. muciniphila* and not receiving FITC-dextran) were also used to estimate the basal fluorescence of mouse serum. Plasma FITC levels were determined by fluorometry using 488 nm for excitation and 520 nm for emission with a microplate reader (Tecan, Lyon, France). The percentage of intestinal permeability was then calculated as the ratio between plasma FITC levels after treatment and before the mice received vehicle or pasteurized *A. muciniphila*.

### Anxiety-related behavioral tests

#### Elevated-plus maze

Anxiety-like behavior was first assessed by the EPM test (ViewPoint Behavior Technology, Lissieu, France) as previously described.^11^ Briefly, the apparatus consisted of two opposite open arms (37 × 6 × 0.6 cm) and two closed arms (37 × 6 × 15 cm), joined by a common central platform (15 × 15 cm) receiving equal illumination (30 lux). The maze was elevated 50 cm above the floor. For the experiment, mice (*n* = 10–12 per group) were acclimatized to the room at least 45 min before the test. The animals were then individually placed in the central zone and allowed to explore the maze for 5 min. They were recorded with a camera, and data were manually scored by a blinded experimenter. The distance covered in the maze was recorded (Ethovision XT 15, Noldus). Anxiety-like behavior was assessed by the number of entries in each arm (considered when the four paws are located within the arm) and time spent in open arms.

#### Hole board test

The hole board test is used to evaluate the rodents’ emotionality, anxiety state, and/or stress responses to an unfamiliar environment.^61^ As described above, mice (*n* = 10–12 per group) were acclimatized to the room at least 45 min before the test and individually placed on one corner of the board facing away from the experimenter.^11^ They were recorded with a camera, and data were manually scored by a blinded experimenter. The number of head dips in the holes, indicative of anxiety-like behavior, was quantified for 5 min.

### Cognitive-related behavioral tests

#### Y-maze

The Y-maze apparatus is a spatial recognition memory test. Mice were placed at the end of one arm and allowed to move freely through the maze for 10 min. Entries into all arms were noted, and a spontaneous alternation was counted if an animal entered three different arms consecutively. The distance covered in the maze was recorded (Ethovision XT 15, Noldus) and data were manually scored by a blinded experimenter. The percentage of spontaneous alternation was calculated according to the formula: [(number of alternations)/(total number of arm entries − 2)] × 100.^62^

#### Novel object recognition (NOR) and novel location recognition (NLR)

The day before testing, mice were placed in an open field arena for 10 min of habituation. On the testing day, they (*n* = 10–12 per group) were placed in the arena containing two identical objects placed on opposite symmetrical corners for an acquisition trial of 10 min. The animals were then removed from their home cage for 1 h and placed again in the arena where one of the familiar objects previously presented had been randomly replaced by a novel object (NOR). The percentage of time spent exploring the novel object was determined. Exploration was defined as the orientation of the animal’s snout toward the object, sniffing or touching with the snout, while running around the object, sitting or climbing on it was not considered as exploration.^63^ After 1 h inter-trial time, animals were retested, while the novel object was relocated in the opposite corner to the familiar object (NLR). The location of novel object *versus* familiar object was counterbalanced. The percentage of time spent exploring the novel object’s location was measured to determine spatial recognition memory. During all the NOR/NLR procedure, mice were recorded with a camera, and data were manually scored by a blinded experimenter.

### Gene expression analysis by RT-qPCR

Total RNAs of proximal colon were extracted with Trizol (ThermoFisher Scientific, Waltham, MS, USA; Cat. No. 15596,026) and reverse-transcribed with a high capacity cDNA RT kit (Applied Biosystems, Waltham, MS, USA; Cat. No. 4368814). Specific cDNA was amplified for Occludin (Forward 5ʹ-AGTACATGGCTGCTGCTGATG-3ʹ; Reverse 5ʹ-CCCACCATCCTCTTGATGTGT-3), Claudin-2 (Forward 5ʹ-ATGCCTTCTTGAGCCTGCTT-3ʹ; Reverse 5ʹ-AAGGCCTAGGATGTAGCCCA-3ʹ), ZO-1 (Forward 5ʹ-GTTCCGGGGAAGTTACGTGC-3ʹ; Reverse 5ʹ-AAGTGGGACAAAAGTCCGGG-3’), and 26S (Forward 5ʹ-TGTCATTCGGAACATTGTAG-3ʹ; Reverse 5ʹ-GGCTTTGGTGGAGGTC-3ʹ). qPCR assays were performed with SsoAdvanced Universal SYBR Green Supermix (Biorad, Hercules, CA, USA; Cat. No. 1,725,271) and carried out on the CFX96 Touch Real-Time PCR Detection System (Biorad, Hercules, CA, USA). Relative quantification of Occludin, Claudin-2, and ZO-1 mRNA levels were expressed as fold-change, using the 2^−ΔΔCt^ method and 26S as housekeeping gene.

To quantify the colonic IL-22 expression, the cDNA was amplified by the EvaGreen ddPCR-based method. Briefly, IL-22 primers (Forward 5ʹ-TGTGCGATCTCTGATGGCTG-3ʹ; Reverse 5ʹ-GCTGGAAGTTGGACACCTCA-3ʹ) were used to prepare a ddPCR reaction mix. It was prepared by consisting of 11 µL of 2X QX200™ ddPCR™ EvaGreen Supermix (Biorad, Hercules, CA, USA), 1.6 µL of 5 µM Fwd/Rev primer mix, 7.4 µL of RNase and DNase free-water and 2 µL of cDNA (corresponding to 5 ng) in order to obtain a final volume of 22 µL. Twenty microliters of the reaction mix were used to generate droplets with the Q × 200droplet generator (Biorad, Hercules, CA, USA). After generation, the droplets were transferred into a 96-well plate, sealed and amplified in a C1000 Thermal Cycler (Biorad, Hercules, CA, USA) under the thermal conditions recommended by the manufacturer: EvaGreen ddPCR-polymerase activation at 95°C for 5 min, 40 cycles of amplification at 95°C for 30 sec (denaturation) and 60°C for 1 min (annealing), droplets stabilization at 4°C for 5 min and 90°C for 5 min followed by an infinite hold at 4°C. A ramp rate of 2°C/sec was used during the amplification steps. EvaGreen ddPCR absolute quantification was done with QX manager software 2.1 (Biorad, Hercules, CA, USA).

### DNA extraction, 16S rRNA gene amplicon sequencing and microbiota analysis

The gut microbiota composition was analyzed from fecal samples collected at the end of the study and kept frozen at −80°C until use. Genomic DNA was extracted with a QIAamp DNA Stool Mini Kit (Qiagen, Hilden, Germany), according to the manufacturer’s instructions, including a bead-beating step. The V4 region of bacterial 16S rRNA gene was amplified using the primers 515F (5’-GTGYCAGCMGCCGCGGTAA-3’) and 806 R (5’-GGACTACNVGGGTWTCTAAT-3’). Purified amplicons were sequenced using a MiSeq following the manufacturer’s guidelines. Sequencing and demultiplexing were performed on MR DNA (www.mrdnalab.com; Shallowater, TX). Sequences were processed using QIIME2 (version 2023.2).^64^ The pipeline included primer removal and denoising using DADA2 to obtain the amplicon sequence variant (ASV) table.^65^ Singletons (ASV present < 2 times) were discarded. Sequences were clustered based on a 0,99% identity, and chimeras were removed using the UCHIME algorithm (implemented in QIIME’s vsearch plugin). Taxonomic classification was performed using a pre-trained naive Bayes classifier implemented in QIIME2 against the SILVA 138 reference database (silva138_AB_V4_classifier.qza).^66^ Reads classified as mitochondria and chloroplast were filtered out, while unassigned ASVs were retained. Taxa that could not be identified on genus-level are referred to the highest taxonomic rank identified. The beta-diversity index (Bray Curtis) was analyzed using Kruskal-Wallis and PERMANOVA statistical tests. Analyses of the taxonomic composition of the microbiota were performed on relative abundance tables of OTUs at the family level using Multiple Mann–Whitney test comparing two groups and using an FDR of 1% in GraphPad Prism version 10.1.1 for Mac.

### Calcium imaging on murine dorsal root ganglia

Total or colon-innervating (L6-S1) DRG from WT or Nav1.8-Cre-TdTomato C57BL6/J male mice were explanted, cleaned of anterior and posterior roots and connective tissue, and digested by an enzyme mix containing collagenase type III (5 mg.mL^−1^, Worthington) and Dispase (10 mg.mL^−1^; Gibco), for 45 min at 37°C. Dorsal root ganglia cell suspensions were centrifuged at 175 g for 15 s after which the supernatant was removed and replaced by 1 mL of Dulbecco’s Modified Eagle Medium (DMEM; SIGMA). The DRG suspension was triturated through fire polished Pasteur pipettes and spun for 5 s at 175 g. The supernatant containing dissociated cells was saved at each trituration step. Trituration was performed nine times using three fire-polish Pasteur pipettes of decreasing diameter. The cell suspension was then centrifuged for 5 min at 175 g. The supernatant was removed and replaced by 600 µL of DMEM supplemented with 10% (v/v) fetal bovine serum, sodium pyruvate (1 mM; Gibco), L-glutamine (2 mM), and penicillin (100 µg.mL^−1^), streptomycin (100 µg.mL-1), vitamins, amino acids (MEM NEAA, Gibco), and NGF (6.25 pg.mL^−1^). The cell suspension was plated on 12 poly-L-Lysine (100 µg.mL^−1^) and laminin (200 µg.mL^−1^) coated glass sheets in culture dishes. The cells were incubated for 30 min at 37°C before addition of 1 mL of DMEM supplemented medium in each well. Cells were incubated at 37°C overnight.

**Figure 8.**
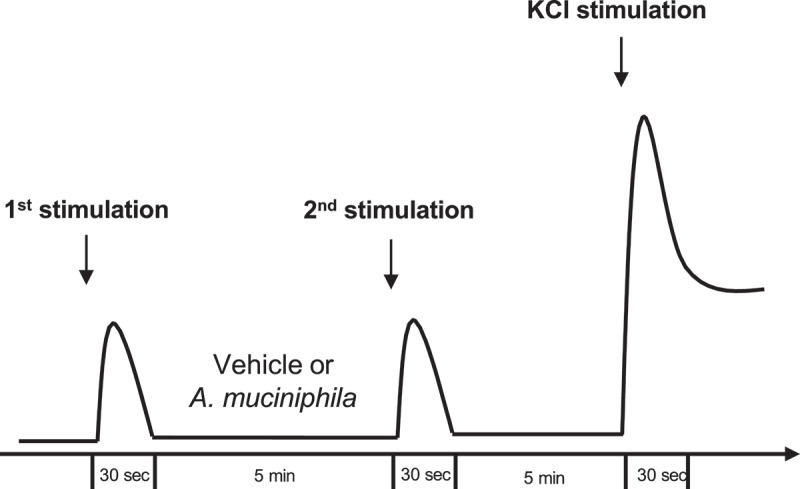
Experimental procedure of *in vitro* evaluation of the neuromodulatory properties of pasteurized *A. muciniphila*. Evaluation of neuronal cell activation by ratiometric calcium imaging on mouse DRG neurons.

On the day of the experiment, intracellular free Ca^2+^ was followed with Ca^2+^ ratiometric dye Fura-2 acetoxymethyl ester (Fura 2-AM, Invitrogen) to assess neuronal cell activation by ratiometric calcium imaging. Briefly, the DRG neurons were loaded with Fura-2 solution (4 µM) supplemented by pluronic acid (1 µg.mL^−1^) for 45 min at 37°C with slight agitation (40 rpm). All imaging experiments were performed in a dark room at temperature. After loading, the glass coverslip was mounted in an imaging/perfusion chamber equipped with a perfusion valve system which was mounted and viewed through an inverted microscope. Neuronal cells were alternately illuminated with 340 nm and 380 nm wavelengths. The exposure time to excitation was 400 ms for each wavelength. Image pairs were acquired every 2 s. All calcium imaging experiments consisted on two stimulations separated by 5 min incubation with pasteurized *A. muciniphila* suspension (1 × 10^9^ TFU/mL) after rinsing with a Tyrode solution (Figure 8). The different stimulations were as follows: (i) KCl (30 mM), (ii) capsaicin (5 µM) (SIGMA, ref. M2028), (iii) inflammatory soap composed by bradykinin (5 µM), histamine (5 µM), serotonin (5 µM), PGE2 (5 µM), Tyrode modified with KCl (10 mM) adjusted at pH6, (iv) serotonin hydrochloride (15 µM) (SIGMA, ref. H9523), (v) bradykinin acetate salt (5 µM) (SIGMA, ref 90,834), and (vi) histamine hydrochloride (15 µM) (SIGMA, ref. H7250). The final KCl (50 mM) depolarization was assessed at the end to evaluate cell viability. The Tyrode solution was composed of NaCl (140 mM), KCl (3 mM), MgCl2 (1 mM), CaCl2 (2 mM), D-Glucose (10 mM), and HEPES (10 mM) adjusted to pH7.4. Osmolarity was also adjusted to 300 mOsm. KCl solution was composed of NaCl (93 mM), KCl (50 mM), MgCl2 (1 mM), CaCl2 (2 mM), D-Glucose (10 mM), and HEPES (10 mM) adjusted to pH7.4. Osmolarity was also adjusted to 300 mOsm.

For data analysis, kinetic stimulation was studied for each viable neuronal cell. The percentage of cells responding to each stimulation before and after vehicle or pasteurized *A. muciniphila* incubation and the fold change in response intensity to the second stimulation (after vehicle or pasteurized *A. muciniphila* incubation) relative to the response intensity to the first stimulation of all viable neuronal cells belonging were calculated.

### Statistical analysis

Statistical analyses were performed with GraphPad Prism 9 software (GraphPad, La Jolla, USA). The results were expressed as mean ± SEM. D’Agostino–Pearson test and Shapiro–Wilk test were used for the normality test. Mann–Whitney and Student’s t-tests were used to compare the two groups. One-way ANOVA, Kruskal–Wallis test, and two-way ANOVA with Tukey’s and Dunn’s multiple post hoc comparison test were used for comparison between more than two groups. Two-Way ANOVA test with a Sidak multiple post hoc comparison test was used for multiple factors influencing. A p value < 0.05 was considered statistically significant.

## Supplementary Material

Supplemental Material

## Data Availability

The raw amplicon sequencing data analyzed in this study have been deposited in the EMBL-EBI European Nucleotide Archive (ENA) under accession number PRJEB53668 (https://www.ebi.ac.uk/ena/browser/view/PRJEB68376). The authors confirm that the data supporting the findings of this study are available within the article and its supplementary materials.
