# Supplementary material for: Pasteurized Akkermansia muciniphila improves irritable bowel syndrome-like symptoms and related behavioral disorders in mice
Source: Gut Microbes. 2024 Jan 3;16(1):2298026. doi: 10.1080/19490976.2023.2298026 (PMC10766393; doi:10.1080/19490976.2023.2298026)
Supplement: Supplemental Material [file KGMI_A_2298026_SM2589.docx]

# SUPPLEMENT FIGURES

# A B C

**5000**

**80** NH

**Intracolonic pressure variation [mmHg.s]**

NMS

**60**

**40**

**20**

**0**

**4000**

**3000**

**Total AUC [mm Hg.s]**

**2000**

**1000**

**0**

# D E F

**5000**

**Total AUC [mm Hg.s]**

**80**

NH NMS

***

*

**Intracolonic pressure variation [mmHg.s]**

**60**

**40**

**20**

**0**

**20 40 60 80**

**80** NH

NMS

**Intracolonic pressure variation [mmHg.s]**

**60**

**40**

**20**

**0**

**4000**

**3000**

**2000**

**1000**

**0**

**Distension Pressure (mmHg)**

**Figure S1. Effects of neonatal maternal separation (NMS) paradigm on colonic hypersensitivity in wild-type (WT) mice.** A NMS paradigm was applied on male and female WT mice. A colorectal distension (CRD) was performed on 8-10 week-old male or 10-12 week-old female non-handled (NH) and NMS mice to identify the mice with colonic hypersensitivity, which were designated as sensitised NMS (sNMS) mice. An NMS mouse was defined as sNMS when its AUC > mean + 2xSD of NH mice AUC. (A and D) Intracolonic pressure variation in response to a colorectal distension in male NH (n=9) and NMS (n=58 and 60) mice. (B and E) Intracolonic pressure variation in response to a colorectal distension in female NH (n=12) and NMS (n= 53 and 51) mice. (C and F) Total area under the curve (AUC) for both male and female NH (n=21) and NMS (n=111 and 112) mice. *p<0.05 ; **p<0.01 ; ***p<0.001 sNMS/Veh vs. sNMS/1.

# A B C

**3000**

✱

**80**

sNMS/Veh sNMS/1 sNMS/5

****

****

**Intracolonic pressure variation [mmHg.s]**

**60**

**80** sNMS/Veh sNMS/1

**60** sNMS/5

**Intracolonic pressure variation [mmHg.s]**

**2000**

**Total AUC [mm Hg.s]**

**40 40**

**20 20**

**0 0**

**20 40 60 80**

**1000**

**0**

**Distension Pressure (mmHg)**

# D E F

**3000** ✱✱

**Total AUC [mm Hg.s]**

**80**

sNMS/Veh sNMS/1 sNMS/5

**

*

**Intracolonic pressure variation [mmHg.s]**

**60**

**80**

**60**

sNMS/Veh sNMS/1 sNMS/5

*

**

**Intracolonic pressure variation [mmHg.s]**

**2000**

**40**

**20**

**0**

**20 40 60 80**

**40**

**20**

**0**

**20 40 60 80**

**1000**

**0**

**Distension Pressure (mmHg) Distension Pressure (mmHg)**

**Figure S2. Effects of pasteurized *A. muciniphila* on colonic hypersensitivity induced in a non- inflammatory IBS mouse model.** The sNMS mice that underwent a neonatal maternal separation paradigm and have developed a colonic hypersensitivity, were treated for 10 days by gavage with a vehicle (sNMS/Veh) or with two different doses (3x109 TFU for the sNMS/1 group or 6x108 TFU for the sNMS/5 group) of pasteurized *A. muciniphila* (pAkk) (n=10 males and n=10 females per group). (A and D) Intracolonic pressure variation in response to a colorectal distension in male sNMS mice. (B and E) Intracolonic pressure variation in response to a colorectal distension in female sNMS mice. (C and F) Total area under the curve (AUC) for both male and female sNMS mice. *p<0,05 ; **p<0.01 ; ****p<0.0001 sNMS/Veh vs. sNMS/1.

# A B ✱


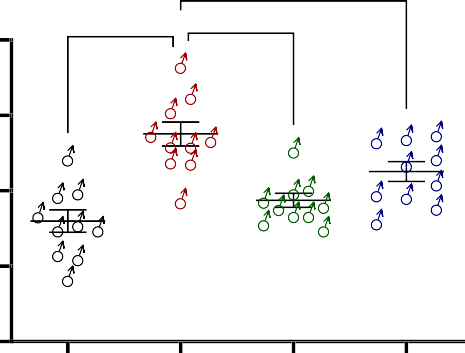


✱✱✱✱

✱✱✱

**40 2000**


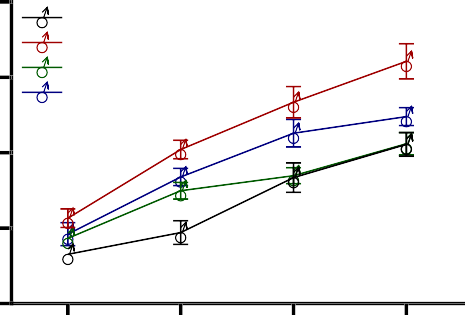


NI/veh Citro/Veh Citro/1 Citro/5

#

*$*$

$ *$*$

***

**Intracolonic pressure variation [mmHg.s]**

**Total AUC [mm Hg.s]**

**30 1500**

**20 1000**

**10 500**

**0**

**20 40 60 80**

**Pressure (mmHg)**

**0**

**NI/Veh Citro/Veh Citro/1 Citro/5**

# C D

**40 2000**


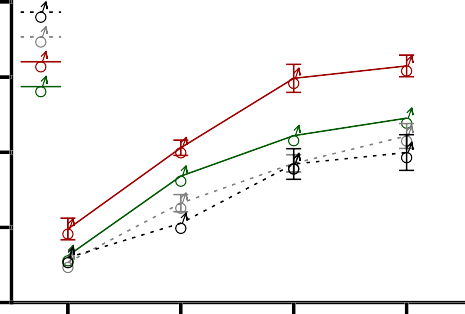


NI/Veh NI/1

Citro/Veh Citro/1

$$

$$

**

**

**

**Intracolonic pressure variation [mmHg.s]**

**Total AUC [mm Hg.s]**

**30 1500**


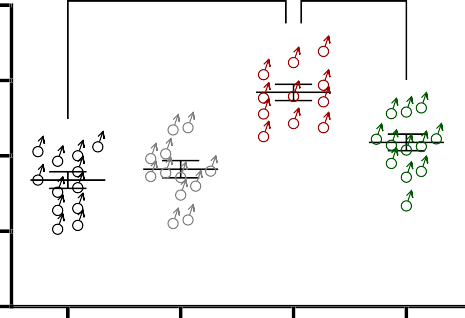
✱✱✱✱ ✱✱✱

**20**

**10**

**0**

**20 40 60 80**

**Pressure (mmHg)**

**Total AUC [mm Hg.s]**

**1000**

**500**

**0**

**NI/Veh NI/1 Citro/Veh Citro/1**

# E F

**40**


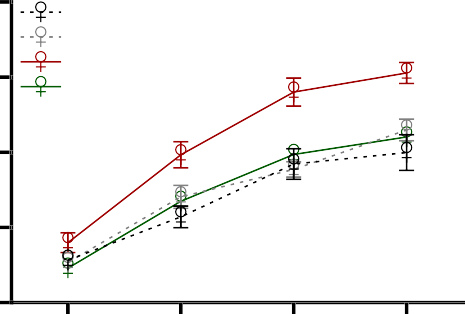


NI/Veh NI/1

Citro/Veh Citro/1

$$

$$

**

**

*$*

**Intracolonic pressure variation [mmHg.s]**

**30**

**20**

**10**

**0**

**20 40 60 80**

**Pressure (mmHg)**

**2500**

**2000**

**1500**

**1000**

**500**

**0**

✱✱✱✱ ✱


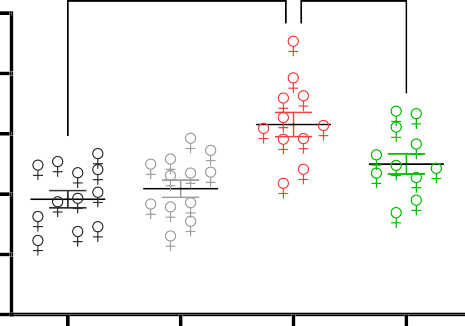


**NI/Veh NI/1 Citro/Veh Citro/1**

**Figure S3. Effects of pasteurized *A. muciniphila* on colonic hypersensitivity induced in a post- infectious IBS mouse model.** Mice were infected with *Citrobacter rodentium* to induce a PI-IBS mouse model. The non-infected mice were inoculated with 200 µL of sterile PBS. During the post-infectious phase, mice were treated by gavage for 8 days from 16 days post infection (DPI) to 23 DPI depending on the group they were included. Non-infected (NI/Veh) and infected (Citro/Veh) mice were forced-fed with the vehicle. Non-infected mice from the NI/1 group were forced-fed with 3x109 TFU of pasteurized *A. muciniphila* (pAkk). Mice from the Citro/1 and Citro/5 group were-forced fed with 3x109 TFU or 6x108 TFU of pasteurized *A. muciniphila*. (A, C and E) Post-infectious colonic sensitivity assessed by measuring intracolonic pressure variations in response to a colorectal distension and (B, D and F) corresponding total area under the curve (AUC) (n=10 per group). * : Citro/Veh vs. NI/Veh. $ : Citro/Veh *vs*. Citro/1. # : Citro/Veh *vs*. Citro/5.
